# Genomic Characteristics of Invasive Mucinous Adenocarcinomas of the Lung and Potential Therapeutic Targets of B7-H3

**DOI:** 10.3390/cancers10120478

**Published:** 2018-11-30

**Authors:** Takahiro Nakagomi, Taichiro Goto, Yosuke Hirotsu, Daichi Shikata, Yujiro Yokoyama, Rumi Higuchi, Sotaro Otake, Kenji Amemiya, Toshio Oyama, Hitoshi Mochizuki, Masao Omata

**Affiliations:** 1 Lung Cancer and Respiratory Disease Center, Yamanashi Central Hospital, Yamanashi 400-8506, Japan; nakagomi.takahiro@gmail.com (T.N.); shikarupd@yahoo.co.jp (D.S.); dooogooodooo@me.com (Y.Y.); r-higuchi1504@ych.pref.yamanashi.jp (R.H.); ootake-bdcg@ych.pref.yamanashi.jp (S.O.); 2 Department of Surgery Keio University, Tokyo 160-8582, Japan; 3 Genome Analysis Center, Yamanashi Central Hospital, Yamanashi 400-8506, Japan; hirotsu-bdyu@ych.pref.yamanashi.jp (Y.H.); amemiya-bdcd@ych.pref.yamanashi.jp (K.A.); h-mochiduki2a@ych.pref.yamanashi.jp (H.M.); m-omata0901@ych.pref.yamanashi.jp (M.O.); 4 Department of Pathology, Yamanashi Central Hospital, Yamanashi 400-8506, Japan; t-oyama@ych.pref.yamanashi.jp; 5 Department of Gastroenterology, University of Tokyo, Tokyo 113-8655, Japan

**Keywords:** lung cancer, invasive mucinous adenocarcinoma, next-generation sequencing, clustering, immunocheckpoint

## Abstract

Pulmonary invasive mucinous adenocarcinoma (IMA) is considered a variant of lung adenocarcinomas based on the current World Health Organization classification of lung tumors. However, the molecular mechanism driving IMA development and progression is not well understood. Thus, we surveyed the genomic characteristics of IMA in association with immune-checkpoint expression to investigate new potential therapeutic strategies. Tumor cells were collected from surgical specimens of primary IMA, and sequenced to survey 53 genes associated with lung cancer. The mutational profiles thus obtained were compared in silico to conventional adenocarcinomas and other histologic carcinomas, thereby establishing the genomic clustering of lung cancers. Immunostaining was also performed to compare expression of programmed death ligand 1 (PD-L1) and B7-H3 in IMA and conventional adenocarcinomas. Mutations in Kirsten rat sarcoma viral oncogene homolog (*KRAS*) were detected in 75% of IMAs, but in only 11.6% of conventional adenocarcinomas. On the other hand, the frequency of mutations in epidermal growth factor receptor (*EGFR*) and tumor protein p53 *(TP53*) genes was 5% and 10%, respectively, in the former, but 48.8% and 34.9%, respectively, in the latter. Clustering of all 78 lung cancers indicated that IMA is distinct from conventional adenocarcinoma or squamous cell carcinoma. Strikingly, expression of PD-L1 in ≥1% of cells was observed in only 6.1% of IMAs, but in 59.7% of conventional adenocarcinomas. Finally, 42.4% and 19.4% of IMAs and conventional adenocarcinomas, respectively, tested positive for B7-H3. Although currently classified as a variant of lung adenocarcinoma, it is also reasonable to consider IMA as fundamentally distinct, based on mutation profiles and genetic clustering as well as immune-checkpoint status. The immunohistochemistry data suggest that B7-H3 may be a new and promising therapeutic target for immune checkpoint therapy.

## 1. Introduction

Invasive mucinous adenocarcinoma (IMA), which represents 2–10% of all lung adenocarcinomas, is considered one of the most malignant subtypes and is associated with poor prognosis [1,2,3]. IMA presents a unique histology among primary lung cancers, and is typified by columnar or goblet cells with basally located nuclei and pale cytoplasm containing varying amounts of mucin [4]. Accordingly, the clinical presentation of IMA is distinct from that of conventional nonmucinous adenocarcinoma [5,6,7]. For example, IMA patients frequently present pneumonia-like symptoms with multifocal and multilobar lesions [5]. Although standard chemotherapy is the only treatment option at advanced stages, no targeted therapy has been demonstrated to be effective against IMA.

IMAs are strongly correlated with mutations in Kirsten rat sarcoma viral oncogene homolog (*KRAS*), which are present in 28–87% of cases [4,5,6,7,8]. However, the correlation between the genetic characteristics and immune-checkpoint expression is unclear and no specific immune checkpoint therapy is established for IMA, although such therapy has recently attracted attention as treatment for non-small cell lung cancer. Molecular studies have been limited and therapeutic targets remain yet to be identified partly because IMA is relatively rare compared to other subtypes. In this study, we surveyed gene mutations in IMA by targeted next-generation sequencing, and propose a novel classification of lung cancers based on clustering of mutational profiles. Furthermore, we investigated by immunohistochemistry the potential of immune checkpoint blockade as therapy against IMA. 

## 2. Results

### 2.1. Patient Characteristics

At first, we enrolled 20 IMA patients and 43 patients with nonmucinous adenocarcinoma (NMA) who underwent surgery at our hospital (Supplementary Table S1). IMA and NMA patients were comparable in age, sex, lung function, smoking habit, tumor location, surgical procedure received, tumor size, pathological stage, and lymphatic or vessel invasion (Table 1, Supplementary Table S1). However, computed tomography revealed that part-solid tumors with ground-glass nodules were more frequent in IMA patients than in NMA patients, and there were no cases of IMA with ground-glass nodules only (Table 1).

### 2.2. Panel Sequencing

**Figure 1:**
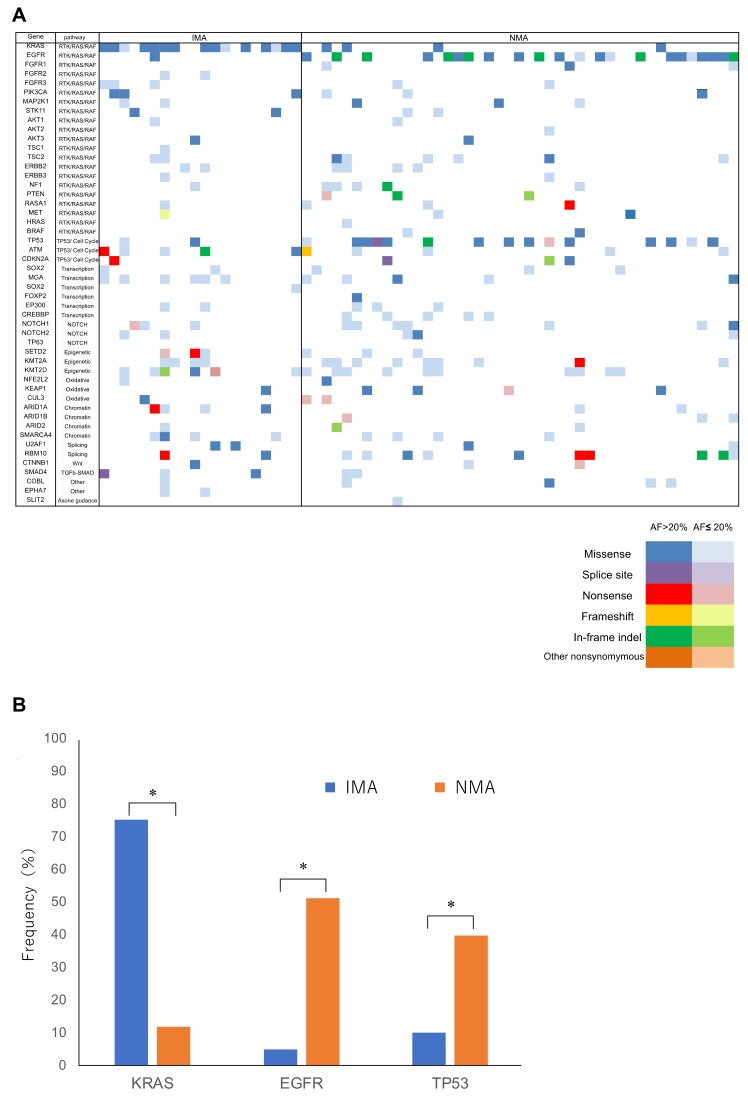
Mutational profile of invasive mucinous adenocarcinoma (IMA) and nonmucinous adenocarcinoma (NMA). (**A**) Most specimens harbored multiple mutations affecting several different functional pathways. However, the prevalence of Kirsten rat sarcoma viral oncogene homolog (*KRAS*), epidermal growth factor receptor (*EGFR*), and tumor protein p53 (*TP53*) mutations was different between IMA and NMA. Nonsynonymous mutations are color-coded as indicated, with dark colors representing allele fractions >20%, and light colors representing allele fractions ≤20%. (**B**) *KRAS* mutations were significantly more frequent in IMA than in NMA. In contrast, mutations in *EGFR* and *TP53* were significantly less frequent in IMA than in NMA. *, *p* < 0.05.

Significant mutations, i.e., those with allele fraction ≥1%, are shown in Figure 1A and listed in Supplementary Table S2 for 20 IMAs and 43 NMAs. Remarkably, *KRAS* mutations were detected in 75% of IMAs (15/20), but only in 11.6% of NMAs (5/43), a statistically significant difference in frequency (Figure 1B). The frequency of mutations in epidermal growth factor receptor (*EGFR*) and tumor protein p53 (*TP53*) were 5% (1/20) and 10% (2/20), respectively, in IMA patients, which are significantly lower rates than the corresponding frequencies of 48.8% (21/43) and 34.9% (15/43) in patients with conventional lung adenocarcinoma (*p* < 0.05, Figure 1B). We note that no significant differences were observed in the mutation burden when the cutoff value was set at allele fraction less than 1% (*p* = 0.82). There were also no significant differences in the distribution of pathways affected in IMAs and NMAs (Supplementary Table S3).

### 2.3. In Silico Analysis

**Figure 2:**
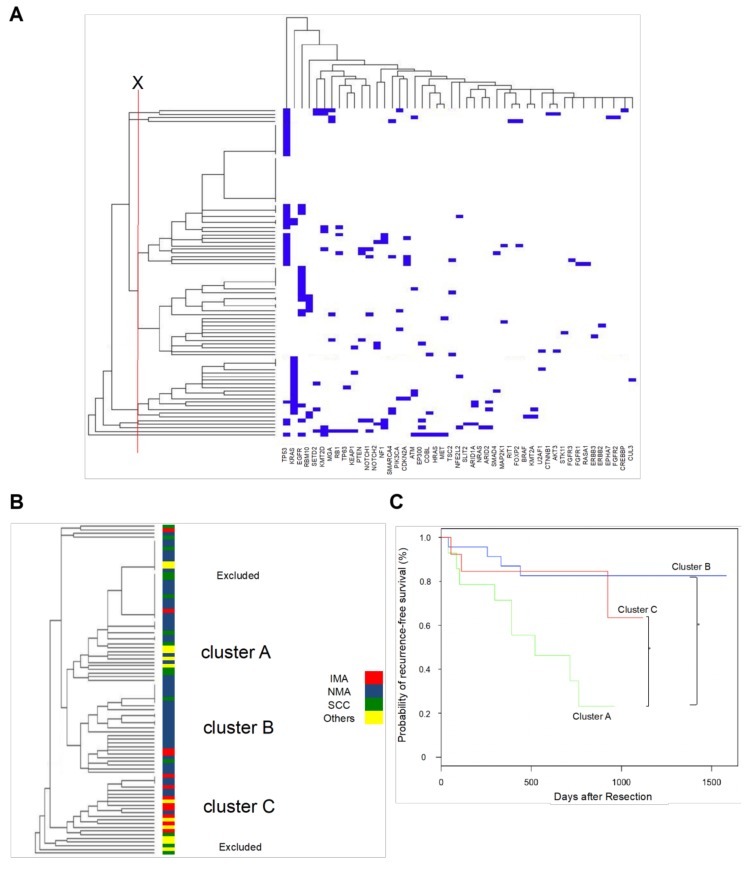
Hierarchical clustering of lung cancer. (**A**) Full view of the cluster diagram. Unsupervised hierarchical clustering was used to group correlated mutations into several clusters, which were assigned based on the threshold marked in red. Results were visualized in TreeView, with mutations on the horizontal axis and cases on the vertical axis. Cases and mutations are arranged such that the most similar are placed next to each other. The length of branches connecting cases or mutations is inversely proportional to profile similarity. (**B**) In this representation, clusters are shown by color-coded dendrogram branches, and conventional histological classifications are superimposed using color-coded bars. Clusters A, B, and C are predominantly squamous cell carcinoma, NMA, and IMA, respectively. (**C**) Recurrence-free survival in individual genomic clusters. Postoperative recurrence-free survival was significantly lower in Cluster A than in Clusters B and C. *, *p* < 0.05.

Mutations obtained by targeted sequencing of specimens from patients with IMA (*n* = 12), NMA (*n* = 43), squamous cell carcinoma (*n* = 13), and other tumors (*n* = 10) were clustered based on similarity by in silico unsupervised hierarchical clustering (Figure 2A). Twelve representative IMA cases were selected out of 20 IMA cases for the hierarchical clustering analysis and the other histological cancers, including squamous cell carcinoma, small cell carcinoma and sarcomatoid cancer, were additionally enrolled as an external control, in order that the inclusion criteria of this analysis might reflect, to some extent, the incidence rate in general of each histological cancer in surgically treated cases (Supplementary Table S1). Results of this analysis were visualized in a dendrogram, in which patients are connected by bars of length proportional to the genetic similarity between them. Upon exclusion of specimens with very few (0–1) mutations detected, as well as a few genetically very remote tumors, most patients were classified into Clusters A, B, and C (Figure 2B).

No significant differences among clusters were observed in age or pathological stage (Table 2), although Cluster A contained significantly more men (*p* = 0.003) and heavy smokers (*p* = 0.008). Importantly, histologic subtypes were unevenly distributed among clusters (Table 2, *p* = 0.001), with 66.7% of squamous cell carcinoma patients grouped in Cluster A, and 80% of IMA cases grouped in Cluster C (Table 2, Figure 2B). In Cluster B, 87.0% of specimens were conventional adenocarcinoma (Table 2, Figure 2B). Patients with other histologic subtypes, including small cell carcinoma and pleomorphic carcinoma, were distributed among Clusters A and C (Table 2).

### 2.4. Correlation between Cluster Classification and Outcome

Postoperative recurrence-free survival was significantly poorer in Cluster A than in Clusters B and C (Figure 2C, log-rank *p* < 0.05, Supplementary Table S1). Based on Cox’s proportional hazards model, pathological stage and cluster are independent risk factors for postoperative recurrence or mortality, whereas sex, age, smoking habit, and histology are not (Table 3). 

### 2.5. Immunohistology for Thyroid Transcription Factor 1 (TTF-1)

For immunohistochemistry study, 13 IMAs and 24 NMAs were additionally enrolled and 33 IMAs and 67 NMAs were examined and compared in total.

**Figure 3:**
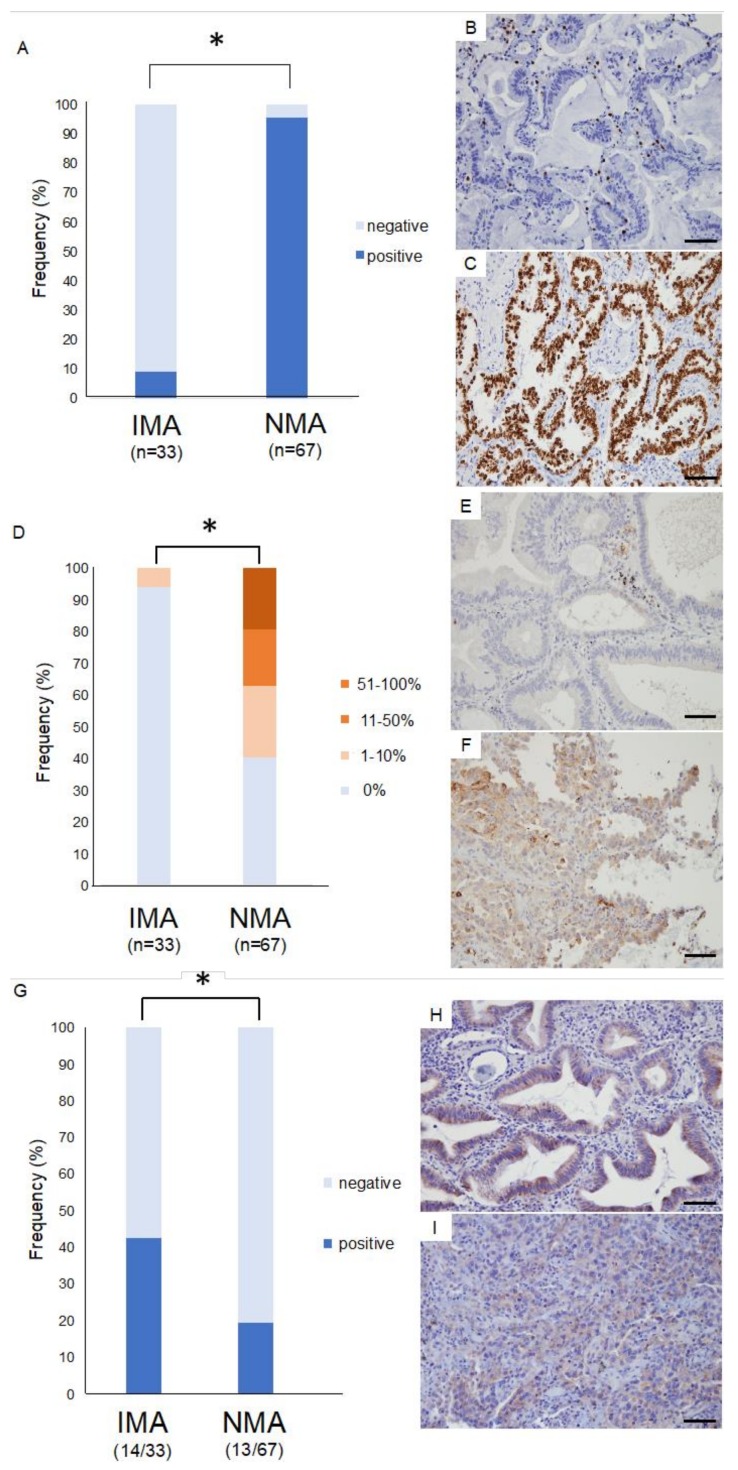
Immunostaining for thyroid transcription factor 1 (TTF-1) and immune checkpoint proteins in invasive mucinous adenocarcinoma (IMA) and nonmucinous adenocarcinoma (NMA). (**A**) TTF-1 was detected in nearly all patients with NMA, but in only a few patients with IMA. *, *p* < 0.05. (**B**,**C**) Representative TTF-1 immunostaining in IMA (**B**) and NMA (**C**). (**D**) Programmed death ligand 1 (PD-L1) was detected in 59.7% patients with nonmucinous adenocarcinoma, but in only 6.1% of patients with invasive mucinous adenocarcinoma, a statistically significant difference in frequency. *, *p* < 0.05. (**E**,**F**) Representative PD-L1 immunostaining in IMA (**E**) and NMA (**F**). (**G**) B7-H3 was detected in 19.4% patients with NMA and in 42.4% of patients with IMA, a statistically significant difference in frequency. *, *p* < 0.05. (**H**,**I**) Representative B7-H3 immunostaining in IMA (**H**) and NMA (**I**). Scale bars: 100 μm.

TTF-1 was detected in only three of 33 IMA specimens (9.1%). This proportion was significantly lower than in NMA specimens, of which 41 of 43 (95.5%) tested positive for TTF-1 (Figure 3A–C, *p* < 0.001).

### 2.6. Immunohistology for Immunocheckpoint Proteins

Immunocheckpoint proteins such as programmed death ligand 1 (PD-L1: B7-H1), V-set domain-containing T-cell activation inhibitor 1 (VTCN1: B7-H4), and B7-H3 were also assayed in formalin-fixed paraffin-embedded specimens. The molecules PD-L1, B7-H3, and VTCN1 belong to a family of immune modulators and have garnered attention as a promising molecular target for immunocheckpoint therapy. PD-L1 was assessed according to a four-tier scale, corresponding to specimens in which the plasma membrane is positively stained in 0%, 1–10%, 11–50%, and 51–100% of cells. Remarkably, only two of 33 IMA specimens (6.1%) tested positive for PD-L1, a positive test being defined as staining of ≥1% of cells. In contrast, a significantly larger number of NMA specimens tested positive (40/67 patients, 59.7%) (Figure 3D–F, *p* < 0.05). VTCN1 was detected in 3 of 13 patients with squamous cell carcinoma (22%), but not in any other patients (Supplementary Figure S1). Finally, B7-H3 was detected in 14 of 33 IMA patients (42.4%). This proportion was significantly higher than in NMA specimens, of which 13 of 67 (19.4%) tested positive for B7-H3 (Figure 3G–I, *p* < 0.05). In addition, signal intensity tends to be stronger in IMA than in NMA (Figure 3H,I).

### 2.7. Association between the Mutation Profiles and Immunocheckpoint Molecules

**Figure 4:**
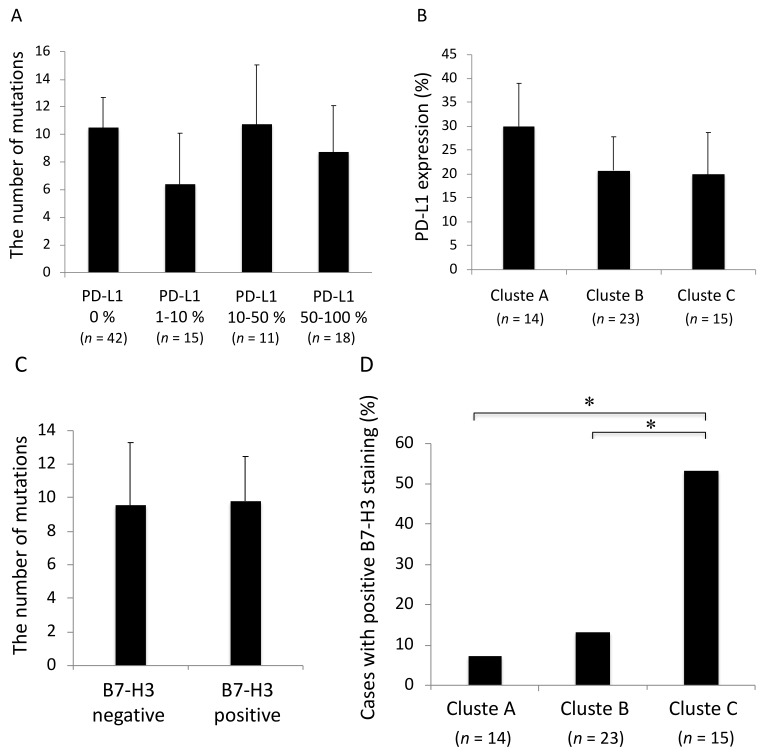
Association between the mutation profiles and PD-L1 expression. (**A**) Estimates of tumor mutation burden and PD-L1 expression. The number of mutations detected by targeted sequencing was not significantly different among the cancers with different PD-L1 expressions (*p* = 0.79). (**B**) Comparison of PD-L1 expression among the genomic clusters of cancer. PD-L1 expression was not significantly different among the clusters A, B and C (*p* = 0.81). (**C**) Estimates of tumor mutation burden and B7-H3 expression. The number of mutations detected by targeted sequencing was not significantly different between the cancers with and without B7-H3 expressions (*p* = 0.86). (**D**) Comparison of B7-H3 expression among the genomic clusters of cancer. B7-H3 expression was significantly elevated in the cluster C compared with the clusters A and B. *, *p* < 0.05.

Based on the aforementioned results, the association between the mutation profiles and immunocheckpoint molecules was examined. Estimates of tumor mutation burden by targeted sequencing did not correlate with PD-L1 or B7-H3 expression (Figure 4A,C, *p* = 0.79, 0.86, respectively). No significant differences of PD-L1 expression were detected among the mutational clusters A, B and C (Figure 4B, *p* = 0.81). Thus, tumor mutation profiles and PD-L1 expression were basically found to be independent variables. On the other hand, B7-H3 expression was significantly elevated in the cluster C compared with the clusters A and B (Figure 4D, *p* < 0.05).

**Figure 5:**
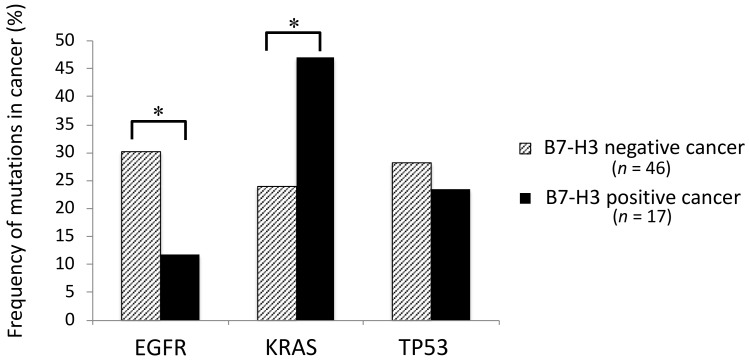
Frequency of *EGFR*, *KRAS,* and *TP53* mutations with or without B7-H3 positivity in adenocarcinoma. There are significant differences of frequency in *EGFR* and *KRAS* mutations between B7-H3 positive and negative adenocarcinomas. *, *p* < 0.05.

*EGFR* mutations were affected significantly more frequently in B7-H3 negative adenocarcinomas than in B7-H3 positive adenocarcinomas, while *KRAS* mutations were affected significantly more frequently in B7-H3 positive adenocarcinomas than in B7-H3 negative adenocarcinomas (Figure 5, *, *p* < 0.05). No significant difference of frequency was found in *TP53* mutations between B7-H3 positive and negative adenocarcinomas (Figure 5).

## 3. Discussion

In this study, we found that lung IMA is genetically distinct from other lung cancers. *KRAS* mutations were the most frequent drivers, while *EGFR* mutations were rare, in line with previous studies [4,5,6,7,8]. *TP53* mutations were similarly rare in IMA, and were detected in only 2 of 20 IMA cases examined, but in approximately 35% of NMA cases. Thus, the mutational profile of IMA is distinct from that of NMA. Accordingly, genomic clustering segregated IMA apart from squamous cell carcinoma and conventional adenocarcinoma. In addition, immunohistology for major immunocheckpoint molecules suggested that, although blockade of programmed death 1 (PD-1) and PD-L1 is unlikely to be effective against IMA, blockade of B7-H3 may prove successful.

Based on the International Association for the Study of Lung Cancer (IASLC)/the American Thoracic Society (ATS)/the European Respiratory Society (ERS) classification, lung adenocarcinomas are either nonmucinous (nonmucinous adenocarcinoma in situ, nonmucinous minimally invasive adenocarcinoma, and NMA) or mucinous (mucinous adenocarcinoma in situ, mucinous minimally invasive adenocarcinoma, IMA, and colloid-predominant adenocarcinoma) [1]. In a survey of 864 surgical cases, Kadota et al. found that 42 (5%) were mucinous, including 1 case of mucinous minimally invasive adenocarcinoma (0.1%), 36 cases of IMA (4%), and 5 cases of colloid-predominant tumors (0.6%) [7]. No mucinous adenocarcinoma in situ was observed [7]. Similarly, mucinous adenocarcinoma in situ was not present in our cohort, which may explain why pure ground-glass opacity was not observed radiologically (Table 1).

In silico analysis suggests some correlation between genomic characteristics and the World Health Organization lung cancer classification. For instance, Cluster A was predominantly composed of squamous cell carcinomas, Cluster B consisted mostly of NMAs, and Cluster C consisted mostly of IMAs. This result suggests that IMA is genetically distinct, although histologic–molecular associations are typically not 100% specific. Nevertheless, cluster classes were significantly associated with postoperative recurrence-free survival, while histologic subtype was not. Thus, hierarchical clustering based on gene mutations is novel, meaningful, and functional classification of lung tumors. Taken together, these findings may explain why IMA is refractory to conventional chemotherapies, and may require different therapeutic strategies.

Although IMA outcomes following surgical resection are relatively favorable, therapeutic outcomes from advanced IMA are extremely poor [2,3]. Thus, a more suitable therapy is currently under development. As IMA tumors lack the major driver mutations present in lung adenocarcinoma, including in *TP53* and *EGFR*, one might expect IMA tumors to respond instead to agents that target *KRAS* mutations. However, *KRAS* itself has proven difficult to inhibit, and the effectiveness of agents that target key *KRAS* effectors is diminished by compensatory or parallel pathways [9,10]. Thus, combinations of the blockade agents are more promising. For example, Manchado et al. recently reported that a combination of MEK and FGFR inhibitors is effective against lung cancers with *KRAS* mutations [11]. Similarly, Kitai et al. reported that combinations of MEK and FGFR inhibitors, may be effective against mesenchymal-like *KRAS*-mutated non-small cell lung carcinoma [12].

Expression of TTF-1, also known as NK2 homeobox-1 (NKX2-1) target protein, is restricted to the lung and thyroid. Accordingly, TTF-1 is frequently used as a marker to distinguish carcinomas of pulmonary and thyroid origin [13]. Of note, loss of TTF-1 and altered differentiation states are associated with IMA, implying that IMA and NMA arise from different cellular lineages [14]. In addition, several studies observed frequent NKX2-1 mutations in IMA, and proposed NKX2-1 as a lineage-specific tumor suppressor in the lung [15,16]. Similarly, mice with *KRAS* mutations and NKX2-1 deletion develop lung tumors that resemble human mucinous lung adenocarcinomas [17,18]. In addition, Guo et al. showed that restoration of TTF-1 expression in mucinous lung cancer cells induces expression of PD-L1 in vitro, suggesting that loss of PD-L1 in IMA may be due to loss of TTF-1 [19]. Collectively, these observations suggest that a specific genomic profile may drive the phenotypic characteristics of IMA, and provide possible routes to therapy.

Immune checkpoints are a suite of costimulatory and inhibitory molecules in T cells that control the amplitude and quality of the immune response [20]. To evade immunity, tumors may overexpress or activate inhibitory immune checkpoints, especially PD-1 and its ligand PD-L1 [21]. While antibodies that inhibit the PD-1/PD-L1 pathway produce a durable clinical response in various solid tumors including non-small cell lung cancer [22,23], they only benefit a fraction of patients. Indeed, our data now suggest that such antibodies are likely to be ineffective against IMA. B7-H3 and B7-H4 (VTCN1) belong to a family of immune modulators that includes PD-L1 (B7-H1) and are regarded as a promising molecular target for immunocheckpoint therapy [24]. In fact, many preclinical studies reported the possible effectiveness of B7-H3- or B7-H4-targeting antibody, among the B7 family, in the treatment of cancers [25,26,27,28,29]. Furthermore, since we would like to elevate our study to translational research, we focused on B7-H3 and B7-H4, which are currently being tested in a clinical trial [30,31,32]. Inamura et al. showed B7-H3 was significantly associated with lung adenocarcinoma in smokers and/or patients with wild type *EGFR* [33]. Our data also showed that B7-H3-positive adenocarcinomas harbor *EGFR* mutations significantly less frequently, suggesting a non-redundant biological role of the two targets: *EGFR* and B7-H3. It was also revealed that B7-H3-expressing cancers harbor *KRAS* mutations significantly more frequently, which may make a breakthrough in the treatment of *KRAS* mutant lung cancers. Moreover, *KRAS* positivity and *EGFR* negativity is a typical genomic pattern of IMA. Notably, approximately 40% of IMA tumors in our study express B7-H3, whereas none of them strongly express PD-L1, indicating that the former is a better immunotherapeutic target in IMA patients than the latter. This finding may open new avenues of treatment based on immune checkpoint inhibitors against proteins other than PD-1/PD-L1.

Proctor et al. reported that B7-H3 expression was elevated in meningiomas harboring gene mutations affecting the AKT pathway [34]. In the case of lung cancer, it is well known that *EGFR* or *KRAS* affects the AKT pathway, whereas wild-type *EGFR* was reported to be associated with B7-H3 expression [33]. i.e., *KRAS* mutation may be the key trigger of B7-H3 expression in lung cancer. In fact, the cases in cluster C, which are mainly related to *KRAS* mutations, expressed B7-H3 most frequently. Importantly, genomic clustering in this study was directly associated with B7-H3 expression, and, thus, distinguished the cancers which can be treated by the B7-H3 blockade therapy.

In our paper, we focused on the relation between the genomic profiles and phenotypic characteristics of IMA. To summarize our data and previous reports, IMA usually harbors both *KRAS* and NKX2-1 mutations, both of which are oncogenic [19]. It is estimated that NKX2-1 mutation leads to loss of TTF-1 and PD-L1 expressions, while *KRAS* mutation tends to upregulate B7-H3 expression—presumably by disrupting the AKT pathway [19,34]. Eventually, B7-H3 can be considered as a new therapeutic target, well specific to IMA.

B7-H3 is a type I transmembrane protein and B7 immunoregulatory molecule of the Ig superfamily [35]. While B7-H3 mRNA is broadly expressed in the human breast, bladder, liver, lung, lymphoid organs, placenta, prostate, and testis [36,37,38], protein expression is low and rare [39]. However, B7-H3 is upregulated in several malignancies including non-small cell lung cancer [40]. In preclinical models, both stimulatory and inhibitory activities have been postulated for B7-H3 in T cell activity against tumors [35,36,37,39]. For example, B7-H3 expression is linked to decreased T cell proliferation and interferon-γ production in human hepatocellular carcinoma [41]. Similarly, B7-H3 blockade increased CD8^+^ T cell proliferation and activity in mouse models of pancreatic cancer [42].

Enoblituzumab, also known as MGA271, is a humanized, Fc-optimized monoclonal antibody against B7-H3, and was shown to be active against a fraction of heavily pretreated solid tumors and was well-tolerated at typical doses in a Phase 1 study [32]. The antibody is reported to have limited activity against normal tissue, and its Fc domain supposedly enhances antitumor activity [32]. The clinical activity of the antibody for B7-H3-expressing cancers is currently under investigation, alone or in combination with monoclonal antibodies against either CTLA-4 or PD-L1 [32]. We anticipate that MGA271 will demonstrate potent antitumor activity against IMA, which expresses B7-H3 but not PD-L1 and is otherwise difficult to treat, in contrast to NMA.

## 4. Methods

### 4.1. Patients and Sample Preparation

The survey covered 123 patients who underwent surgery for lung cancer in our department between June 2014 and June 2018. We obtained written informed consent from these patients for genetic research, in accordance with protocols approved by the Institutional Review Board at our hospital. Specimens were typed histologically according to World Health Organization classification (third edition) [43], and staged according to International Union Against Cancer TNM classification (eighth edition) [44]. In total, 86 cancers were subject to the mutation analysis, which included 20 IMA, 43 conventional adenocarcinomas, 13 squamous cell carcinomas, and 10 other histological cancers. Of these 20 IMA, 16 were pure mucinous, and 4 were mixed mucinous/nonmucinous. Serial sections of formalin-fixed, paraffin-embedded tissue were stained with hematoxylin-eosin and microdissected using an ArcturusXT laser-capture microdissection system (Thermo Fisher Scientific, Tokyo, Japan). DNA was extracted by QIAamp DNA FFPE Tissue Kit (Qiagen, Tokyo, Japan), and DNA quality was checked using primers against ribonuclease P. A peripheral blood sample was also drawn from each patient just before surgery. The blood sample was centrifuged, and DNA was extracted from the buffy coat by utilizing QIAamp DNA Blood Mini Kit (Qiagen).

### 4.2. Targeted Deep Sequencing and Data Analysis

A panel of exons in 53 genes (see Supplementary Table S4) was established based on (a) frequent association with lung cancer, as reported in TCGA [45,46] and other surveys [47,48,49,50,51], and (b) frequent mutation in lung cancer, as reported in COSMIC, Catalogue of Somatic Mutations In Cancer [52] Primers for panel sequencing were designed in Ion AmpliSeq (Thermo Fisher Scientific) as we previously reported [53,54,55,56,57,58,59]. Sequencing libraries were made by Ion AmpliSeq Library Kit (Thermo Fisher Scientific). After barcoding with Ion Xpress Barcode Adapters (Thermo Fisher Scientific), libraries were purified using Agencourt AMPure XP (Beckman Coulter, Tokyo, Japan) and quantified by Ion Library Quantitation Kit (Thermo Fisher Scientific). After being templated with Ion PI Template OT2 200 Kit v3 (Thermo Fisher Scientific), the libraries were sequenced on an Ion Proton (Ion Torrent) using Ion PI Sequencing 200 Kit v3.

Raw signal data were analyzed in Torrent Suite version 4.0, and processed by standard Ion Torrent Suite Software. The pipeline was composed of signal processing, base calling, quality score assignment, read alignment to human genome 19, quality control of mapping, and coverage analysis. Single nucleotide variants, insertions, and deletions were then annotated by tumor–normal pair analysis against lymphocytes from peripheral blood, using Ion Reporter Server System (Thermo Fisher Scientific). Results were visualized with Integrative Genomics Viewer (Broad institute, Boston, MA, USA).

### 4.3. In Silico Clustering

Hierarchical clustering generates a binary tree, in which the most similar patterns are clustered. To organize tumor mutational profiles into meaningful structures based on similarity or dissimilarity, we used unsupervised hierarchical clustering with average and complete linkage algorithms in GeneCluster, applying the same approach as previously reported [60,61,62,63]. This process places cases with similar mutation profiles as neighboring rows in the clustergram. Relationships between cases were then visualized as a dendrogram with branch length inversely proportional to the similarity between mutational profiles. Only mutations with allele fraction more than 1.0% were included in this analysis. Results were visualized with TreeView [64].

### 4.4. Immunohistochemistry

Formalin-fixed paraffin-embedded tissues were sectioned at 5 μm, deparaffinized, rehydrated, and stained in an automated system (Ventana Benchmark ULTRA system; Roche, Tucson, AZ, USA) using commercially available detection kits and 1:250 dilutions of antibodies against TTF-1 (SPT24; Biocare), PD-L1 (28–8) (ab205921; Abcam, Cambridge, UK), VTCN1 (D1M8I; Cell Signaling Technology, Danvers, MA, USA), and B7-H3 (D9M2L; Cell Signaling Technology). All slides were stained within two months post-sectioning. The cutoff for positive staining of TTF-1, B7-H3, and VTCN1 was >1% at any intensity, and samples were dichotomized as positive or negative. For PD-L1, expression was evaluated by two pathologists on a quantitative scale from 0% to 100%.

### 4.5. Statistical Analyses

Continuous variables are reported as means and standard deviations. Categorical variables were compared by chi-squared test. To determine predictors of recurrence-free survival within the cohort, we constructed Cox proportional hazards models based on variables of interest. Recurrence-free survival was defined as the period from the day of operation to the day of recurrence or the day of final follow-up. Survival was assessed by the Kaplan–Meier method, and survival curves were compared by log-rank test. Multivariate analyses and calculations of hazard ratios and 95.0% confidence intervals were performed in JMP (SAS Institute, Cary, NC, USA). Two-tailed *p* < 0.05 denoted statistically significant difference.

## 5. Conclusions

Based on our analysis of mutation profiles and genetic clustering, IMA is found to be a distinct entity from nonmucinous adenocarcinoma. Although IMA is considered to be difficult to treat, the immunohistochemistry data suggest that immunotherapy against B7-H3 may prove successful. Further studies are needed to validate our findings and to make them applicable to the clinical settings.

## Figures and Tables

**Table 1:** Patient characteristics.

Characteristic	IMA (*n* = 20)	NMA (*n* = 43)	*p* Value
Age	72.7 ± 7.9	67.9 ± 8.3	0.98
Sex			
Male	12 (60%)	27 (62.8%)	0.83
Female	8 (40%)	16 (37.2%)	
Performance Status			
0	17 (85%)	36 (83.7%)	
1	3 (15%)	7 (16.3%)	0.90
≧2	0 (0%)	0 (0%)	
Smoking habit			
Never	10 (50%)	15 (34.9%)	0.26
Former/current	10 (50%)	28 (65.1%)	
Smoking index	383 ± 107	476 ± 86	0.26
CT finding			
Solid	9 (45%)	20 (46.5%)	
Part solid GGN	11 (55%)	8 (18.6%)	0.0002
GGN	0 (0%)	15 (34.9%)	
Tumor location			
Central	0 (0%)	0 (0%)	
Middle	7 (35%)	8 (18.6%)	0.16
Peripheral	13 (65%)	35 (81.4%)	
Surgical procedure received			
Sublobar resection	1 (5%)	10 (23.3%)	
Lobectomy	18 (90%)	30 (69.8%)	0.15
Pneumonectomy	1 (5%)	1 (2.3%)	
Other	0 (0%)	2 (4.6%)	
Tumor size (mm)	37.2 ± 6.8	20.7 ± 1.4	1.00
Pathological stage			
I	17 (85%)	38 (88.4%)	
II	3 (15%)	1 (2.3%)	0.10
III	0 (0%)	3 (7.0%)	
IV	0 (0%)	1 (2.3%)	
Pathological lymphatic invasion			
Absent	18 (90%)	37 (86.5%)	0.66
Present	2 (10%)	6 (13.5%)	
Pathological vessel invasion			
Absent	17 (85%)	33 (76.7%)	
Microscopically present	3 (15%)	10 (23.3%)	0.44
Macroscopically present	0 (0%)	0 (0%)	

IMA, invasive mucinous adenocarcinoma; NMA, nonmucinous adenocarcinoma; CT, computed tomography; GGN, ground-glass nodule. Peripheral, central, and middle lung cancers correspond to primary lesions located in the outer, inner, or middle one-third of the lung field, respectively. Pathological staging was performed according to the International Union Against Cancer tumor–node–metastasis classification (eighth edition).

**Table 2:** Characteristics of genomic clusters.

Characteristic		Cluster A	Cluster B	Cluster C	*p* Value
		*n* = 14	*n* = 23	*n* = 15	
Sex					0.003
	male	14 (100%)	13 (56.5%)	9 (60%)	
	female	0 (0%)	10 (43.5%)	6 (40%)	
Age		70.4 ± 8.7	70.2 ± 7.9	70.6 ± 8.0	0.952
Smoking index					0.008
	0	0	10	5	
	1–1000	7	10	6	
	1000<	7	3	4	
Histology					0.001
	NMA	6	20	4	
	IMA	0	2	8	
	SCC	4	1	1	
	other	4	0	2	
Pathological stage					0.158
	0-IA	4	15	8	
	IB	4	6	3	
	IIA-IV	6	2	4	

IMA, invasive mucinous adenocarcinoma; NMA, nonmucinous adenocarcinoma; SCC, squamous cell carcinoma.

**Table 3:** Multivariate proportional hazard model of risk factors for postoperative recurrence or mortality.

Variables	Hazard ratio (95% CI)	*p* Value
Cluster		
cluster A	1 (Ref.)	
cluster B	0.25 (0.05–0.94)	0.04
cluster C	0.18 (0.03–0.78)	0.02
Pathological stage		
stage 0 or IA	1 (Ref.)	
stage IB	22.58 (1.80–881.50)	0.01
stage IIA or more	36.09 (2.83–1972.69)	0.003
Male (ref. Female)	1.25 (0.24–9.21)	0.75
Age		
–65	1 (Ref.)	
66–75	1.08 (0.34–7.36)	0.71
76–	1.22 (0.56–8.31)	0.55
Smoker (ref. non-smoker)	1.56 (0.61–8.54)	0.74
Histology		
NMA	1 (Ref.)	
IMA	0.46 (0.01–7.14)	0.60
SCC	6.94 (0.34–163.65)	0.20
Others	3.05 (0.05–295.07)	0.59

NMA, nonmucinous adenocarcinoma; IMA, invasive mucinous adenocarcinoma; SCC, squamous cell carcinoma; Ref., reference.

## Supplementary Materials

The following are available online at http://www.mdpi.com/2072-6694/10/12/478/s1, Figure S1: Immunostaining for VTCN-1 in IMA, NMA, and squamous cell carcinoma, Table S1: Clinical characteristics of the patients, Table S2: Mutations in IMA specimens, Table S3: Pathways affected in IMA and NMA by mutations with allele fractions ≥20%, Table S4: Sequencing targets.
