# Supplementary material for: Genomic Characteristics of Invasive Mucinous Adenocarcinomas of the Lung and Potential Therapeutic Targets of B7-H3
Source: Cancers (Basel). 2018 Nov 30;10(12):478. doi: 10.3390/cancers10120478 (PMC6316015; doi:10.3390/cancers10120478)
Supplement: Supplementary file 1 [file cancers-10-00478-s001.pdf]

Supplementary Materials: Genomic Characteristics of Invasive Mucinous Adenocarcinomas of the Lung and Potential Therapeutic Targets of B7-H3

Takahiro Nakagomi, Taichiro Goto, Yosuke Hirotsu, Daichi Shikata, Yujiro Yokoyama, Rumi Higuchi, Sotaro Otake, Kenji Amemiya, Toshio Oyama, Hitoshi Mochizuki, and Masao Omata

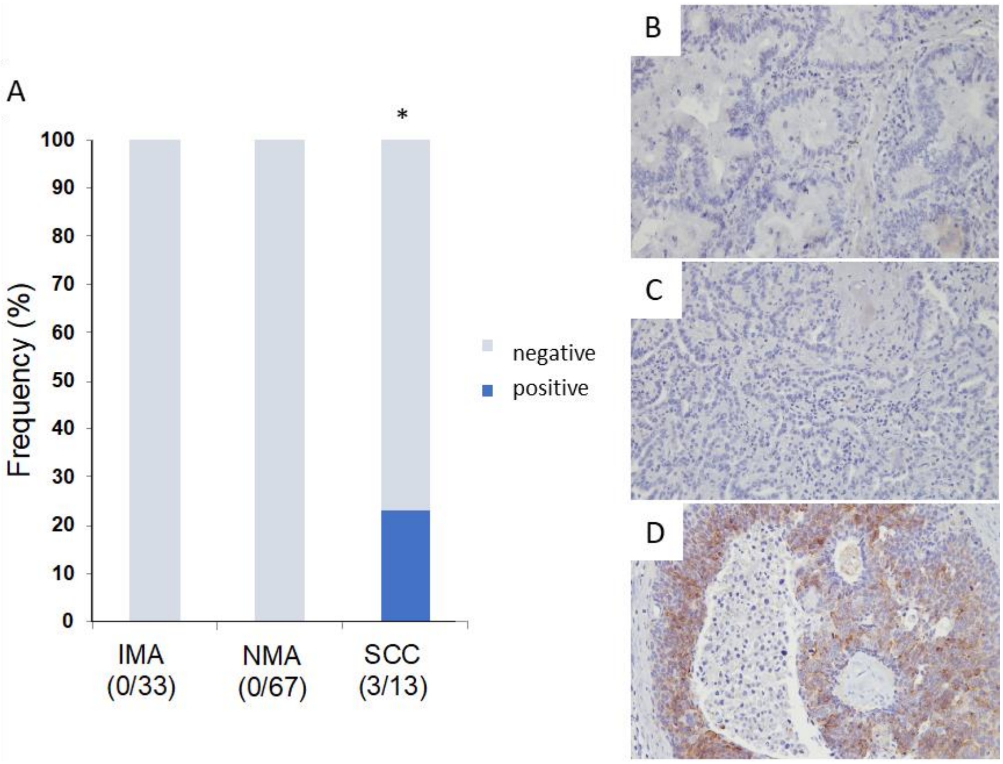

Figure S1. Immunostaining for VTCN-1 in IMA, NMA, and squamous cell carcinoma. (**A**) V-set domain-containing T-cell inhibitor 1 (VTCN1) was detected in some patients with squamous cell carcinoma, but not in patients with invasive mucinous adenocarcinoma (IMA) or nonmucinous adenocarcinoma (NMA). *, $p < 0.05$, compared to IMA or NMA. (**B–D**) Representative VTCN1 immunostaining in IMA (B), NMA (**C**), and squamous cell carcinoma (**D**).

Table S1. Clinical characteristics of the patients.

Case	Histology	Cluster analysis	Cluster	Age	sex	Smoking	Brinkman index	Tumor size(mm)	T-N-M	p-Stage	Surgical procedure	Adjuvant Tx	Outcome
1	IMA	Performed	B	67	female	never	0	32	2a-0-0	IB	lobectomy	-	24m no rec
2	IMA	Performed	B	74	male	past	1080	10	1a-0-0	IA1	lobectomy	-	33m no rec
3	IMA	Performed	C	70	male	past	760	22	1c-0-0	IA3	lobectomy	-	33m no rec
4	IMA	Performed	C	74	female	never	0	11	1b-0-0	IA2	wedge resection	-	22m no rec
5	IMA	Performed	C	71	male	past	200	11	1b-0-0	IA2	lobectomy	-	26m no rec
6	IMA	Performed	C	83	female	never	0	20	1b-0-0	IA2	lobectomy	-	19m no rec
7	IMA	Performed	C	72	female	never	0	31	2a-0-0	IB	lobectomy	+	14m no rec
8	IMA	Performed	C	63	male	never	0	80	4-0-0	IIIA	lobectomy	+	31m no rec
9	IMA	Performed	C	49	female	never	0	40	2a-0-0	IB	lobectomy	+	37m no rec
10	IMA	Performed	C	70	male	past	1200	10	1a-0-0	IA1	lobectomy	-	13m no rec
11	IMA	Performed	excluded	74	male	current	600	50	2b-0-0	IIA	lobectomy	-	33m no rec
12	IMA	Performed	excluded	76	male	past	60	10	1a-0-0	IA1	lobectomy	-	14m no rec
13	IMA	-	-	77	female	never	0	28	1c-0-0	IA3	lobectomy	-	6m no rec
14	IMA	-	-	73	male	past	1200	60	2b-0-0	IIA	lobectomy	+	10m no rec
15	IMA	-	-	71	female	never	0	25	1c-0-0	IA3	lobectomy	-	36m no rec
16	IMA	-	-	68	male	past	960	15	1b-0-0	IA2	lobectomy	-	20m no rec
17	IMA	-	-	90	male	past	1000	30	1c-0-0	IA3	lobectomy	-	6m death
18	IMA	-	-	84	female	never	0	90	4-0-0	IIIA	lobectomy	-	1m death
19	IMA	-	-	69	male	current	600	120	4-0-0	IIIA	pneumonectomy	+	18m rec
20	IMA	-	-	78	male	never	0	50	2a-0-0	IB	lobectomy	+	24m no rec
21	Ad	Performed	A	67	male	past	580	20	2a-0-1a	IV	lobectomy	+	32m no rec
22	Ad	Performed	A	74	male	current	1000	35	2a-0-0	IB	lobectomy	+	9m death
23	Ad	Performed	A	58	male	current	340	28	1b-2-0	IIIA	lobectomy	+	30m no rec
24	Ad	Performed	A	49	male	past	400	15	1a-0-0	IA	lobectomy	-	24m no rec
25	Ad	Performed	A	60	male	past	990	12	2b-3-0	IIIB	wedge resection	+	12m rec
26	Ad	Performed	A	73	male	current	1100	20	2a-0-0	IB	wedge resection	-	15m noi rec
27	Ad	Performed	B	63	female	never	0	25	1b-0-0	IA	lobectomy	-	34m no rec
28	Ad	Performed	B	75	female	never	0	20	1a-0-0	IA	lobectomy	-	32m no rec
29	Ad	Performed	B	61	female	never	0	14	1a-0-0	IA	wedge resection	-	17m rec
30	Ad	Performed	B	54	female	never	0	16	1a-2-0	IIIA	lobectomy	+	7m rec
31	Ad	Performed	B	75	male	current	825	12	1a-0-0	IA	lobectomy	-	30m no rec
32	Ad	Performed	B	67	male	past	800	32	2a-0-0	IB	lobectomy	-	1m death
33	Ad	Performed	B	59	female	past	700	35	2a-0-0	IB	lobectomy	+	29m no rec
34	Ad	Performed	B	70	female	never	0	35	2a-0-0	IB	lobectomy	-	29m no rec
35	Ad	Performed	B	82	male	past	150	20	1b-0-0	IA	lobectomy	-	27m no rec
36	Ad	Performed	B	55	male	past	700	17	1a-0-0	IA	segmentectomy	-	25m no rec
37	Ad	Performed	B	72	male	past	300	10	1a-0-0	IA	segmentectomy	-	23m no rec
38	Ad	Performed	B	72	male	past	150	10	is-0-0	0	wedge resection	-	23m no rec
39	Ad	Performed	B	74	female	never	0	8	1a-0-0	IA	wedge resection	-	22m no rec
40	Ad	Performed	B	68	female	never	0	10	1a-0-0	IA	lobectomy	-	24m no rec
41	Ad	Performed	B	64	male	never	0	35	2a-0-0	IB	lobectomy	+	25m no rec
42	Ad	Performed	B	63	male	past	740	25	1a-0-0	IA	lobectomy	-	23m no rec
43	Ad	Performed	B	85	female	never	0	30	2a-0-0	IB	lobectomy	+	24m no rec
44	Ad	Performed	B	73	male	past	1800	8	is-0-0	0	pneumonectomy	+	16m no rec
45	Ad	Performed	B	78	male	past	330	15	1a-0-0	IA	lobectomy	-	16m no rec
46	Ad	Performed	B	75	male	past	800	17	is-0-0	0	lobectomy	-	18m no rec
47	Ad	Performed	C	77	male	current	1120	35	2a-0-0	IB	lobectomy	-	33m no rec
48	Ad	Performed	C	67	male	past	500	15	1a-0-0	IA	wedge resection	+	32m no rec
49	Ad	Performed	C	71	male	current	800	10	1a-0-0	IA	lobectomy	-	2m death
50	Ad	Performed	C	69	male	past	2520	20	2a-1-0	IIA	lobectomy	+	22m no rec
51	Ad	Performed	Excluded	63	female	never	0	4	is-0-0	0	wedge resection	-	34m no rec
52	Ad	Performed	Excluded	59	female	never	0	25	2a-0-0	IB	lobectomy	-	15m rec
53	Ad	Performed	Excluded	74	male	current	800	10	1a-0-0	IA	lobectomy	+	32m no rec
54	Ad	Performed	Excluded	78	female	never	0	10	1a-0-0	IA	lobectomy	-	30m no rec
55	Ad	Performed	Excluded	64	female	past	172	25	1a-0-0	IA	lobectomy	-	29m no rec
56	Ad	Performed	Excluded	49	female	never	0	15	1a-0-0	IA	lobectomy	-	29m no rec
57	Ad	Performed	Excluded	82	male	past	550	25	1b-0-0	IA	lobectomy	-	27m no rec
58	Ad	Performed	Excluded	55	male	past	525	32	1b-0-0	IA	lobectomy	-	25m no rec
59	Ad	Performed	Excluded	71	male	current	1020	30	2a-0-0	IB	lobectomy	+	4m rec
60	Ad	Performed	Excluded	65	male	past	360	25	1a-0-0	IA	segmentectomy	-	24m no rec
61	Ad	Performed	Excluded	79	male	past	1640	13	is-0-0	0	lobectomy	-	13m no rec
62	Ad	Performed	Excluded	67	female	never	0	20	1a-0-0	IA	lobectomy	-	17m no rec
63	Ad	Performed	Excluded	67	female	never	0	25	2a-0-0	IB	lobectomy	-	17m no rec
64	Sq	Performed	A	68	male	past	1860	20	1a-0-0	IA	lobectomy	-	32m no rec
65	Sq	Performed	A	82	male	past	1100	60	2b-1-0	IIA	lobectomy	-	24m death
66	Sq	Performed	A	73	male	past	1800	33	2a-0-0	IB	pneumonectomy	+	16m no rec
67	Sq	Performed	A	77	male	past	675	20	2b-1-0	IIB	lobectomy	+	14m no rec
68	Sq	Performed	B	78	male	current	1700	60	2a-2-0	IIIA	lobectomy	-	11m death
69	Sq	Performed	C	79	male	past	1640	27	1b-0-0	IA	lobectomy	-	13m no rec
70	Sq	Performed	Excluded	72	male	current	1500	140	3-2-0	IIIA	lobectomy	-	1m death
71	Sq	Performed	Excluded	81	male	past	870	35	2a-0-0	IB	lobectomy	-	31m no rec
72	Sq	Performed	Excluded	57	male	past	1060	60	2b-1-0	IIB	lobectomy	+	29m no rec
73	Sq	Performed	Excluded	57	male	past	1060	15	2a-0-0	IB	wedge resection	+	29m no rec
74	Sq	Performed	Excluded	79	female	never	0	90	3-0-0	IIB	lobectomy	-	12m death
75	Sq	Performed	Excluded	69	male	past	920	35	3-0-0	IIB	lobectomy	+	19m death
76	Sq	Performed	Excluded	75	male	current	1080	32	3-0-0	IIB	lobectomy	+	23m no rec
77	small	Performed	A	77	male	past	675	20	2a-0-0	IB	lobectomy	+	12m rec
78	small	Performed	Excluded	64	male	past	1200	35	2a-0-0	IB	lobectomy	+	29m no rec
79	small	Performed	Excluded	73	male	current	1100	18	2a-0-0	IB	lobectomy	-	13m no rec
80	other	Performed	Excluded	68	male	past	90	35	2a-0-1a	IV	lobectomy	+	31m no rec
81	other	Performed	A	83	male	current	2520	110	3-0-0	IIB	lobectomy	-	4m death
82	other	Performed	A	67	male	past	1800	40	2a-2-0	IIIA	Incisional biopsy	+	2m rec
83	other	Performed	C	54	male	current	600	50	2a-0-1a	IV	lobectomy	+	4m death
84	other	Performed	C	78	female	current	780	47	3-0-0	IIB	lobectomy	-	19m no rec
85	other	Performed	Excluded	60	female	never	0	30	1b-0-0	IB	lobectomy	-	23m rec
86	other	Performed	A	74	male	past	1350	28	1b-0-0	IA	lobectomy	-	29m death

Table S2. Mutations in IMA specimens.

Patients	Gene	Function	Protein	Position	Reference	Tumor Variant	allele fraction
Case 1	CTNNB1	Wnt	p.Leu427Phe	chr3:41275113	C	T	72%
	TP53	TP53/ Cell Cycle	p.Gly115Val	chr17:7578469	C	A	69%
	KMT2D	Epigenetic	p.Pro3145Ser	chr12:49431706	G	A	27%
	AKT3	RTK/RAS/RAF	p.Trp410Cys	chr1:243708833	C	A	26%
	SETD2	Epigenetic	p.Gln97Ter	chr3:47165837	G	A	22%
Case 2	KRAS	RTK/RAS/RAF	p.Gly12Val	chr12:25398284	C	A	35%
	ATM	TP53/ Cell Cycle	p.Ile2629fs	chr11:108203577	CTTATA	C	34%
Case 3	U2AF1	Splicing	p.Ser34Phe	chr21:44524456	G	G/A	33%
	KRAS	RTK/RAS/RAF	p.Gly12Asp	chr12:25398280	GCCAC	GCCAC/GCCAT	32%
	KMT2D	Epigenetic	p.Arg2645Ter	chr12:49433620	G	G/A	6%
	SOX2	Transcription	p.Met294Ile	chr3:181431030	G	G/A	4%
Case 4	SMAD4	TGFb−SMAD	splicesite_3	chr18:48591977	G	G/A	54%
	KRAS	RTK/RAS/RAF	p.Gly12Arg	chr12:25398280	GCCACC	GCCACC/GCCACG	44%
	ATM	TP53/ Cell Cycle	p.Gln201Ter	chr11:108114784	C	C/T	40%
	FGFR3	RTK/RAS/RAF	p.Ala636Thr	chr4:1807841	G	G/A	12%
	SOX2	Transcription	lp.Met294Ile	chr3:181431030	G	G/A	6%
	MGA	Transcription	p.Leu28Val	chr15:41961174	T	T/G	4%
Case 5	KRAS	RTK/RAS/RAF	p.Gly12Ala	chr12:25398280	GCCAC	GCCAC/GCCAG	35%
	CDKN2A	TP53/ Cell Cycle	p.Trp15Ter	chr9:21974782	C	C/T	26%
	PIK3CA	RTK/RAS/RAF	p.His1047Leu	chr3:178952085	A	A/T	25%
	FGFR3	RTK/RAS/RAF	p.Ala636Thr	chr4:1807841	G	G/A	8%
Case 6	PIK3CA	RTK/RAS/RAF	p.His1047Leu	chr3:178952085	A	A/T	39%
	NFE2L2	Oxidative	p.Leu30Phe	chr2:178098957	G	G/A	18%
	KRAS	RTK/RAS/RAF	p.Gly12Asp	chr12:25398280	GCCAC	GCCAC/GCCAT	18%
	MAP2K1	RTK/RAS/RAF	p.Lys168Arg	chr15:66735682	A	A/G	11%
	TP53	TP53/ Cell Cycle	p.Arg280Thr	chr17:7577094	GGTCTCT	GTCTCT/GGTCTG	9%
	ATM	TP53/ Cell Cycle	p.Glu953Lys	chr11:108141809	G	G/A	8%
	NOTCH2	NOTCH	p.Ile1689Phe	chr1:120465007	T	T/A	7%
	KMT2D	Epigenetic	p.Asp632Glu	chr12:49445570	G	G/C	6%
	MGA	Transcription	p.Arg3038Trp	chr15:42059392	C	C/T	5%
Case 7	STK11	RTK/RAS/RAF	p.Pro221Leu	chr19:1220644	C	C/T	37%
	NOTCH1	NOTCH	p.Glu794Ter	chr9:139407560	C	C/A	5%
Case 8	CUL3	Oxidative	p.Ser585Cys	chr2:225360637	G	G/C	23%
	KRAS	RTK/RAS/RAF	p.Gly12Asp	chr12:25398280	GCCAC	GCCAC/GCCAT	23%
	NOTCH1	NOTCH	p.Gly1434Ser	chr9:139400048	C	C/T	5%
Case 9	KRAS	RTK/RAS/RAF	p.Gly12Cys	chr12:25398280	GCCACC	GCCACC/GCCACA	71%
	EGFR	RTK/RAS/RAF	p.Ala16Val	chr7:55087017	C	C/T	23%
	ARID1A	Chromatin	p.Trp1844Ter	chr1:27105920	G	G/A	22%
	AKT1	RTK/RAS/RAF	p.Cys296Gly	chr14:105239659	A	A/C	8%
	FGFR3	RTK/RAS/RAF	p.Gly192Asp	chr4:1803223	G	G/A	6%
	KMT2D	Epigenetic	p.Asp1749Glu	chr12:49437723	A	A/C	6%
	SMARCA4	Chromatin	p.Leu724Phe	chr19:11121103	C	C/T	4%
	TSC2	RTK/RAS/RAF	p.Ala1238Thr	chr16:2131697	G	G/A	4%
	KRAS	RTK/RAS/RAF	p.Gly12Cys	chr12:25398280	GCCACC	GCCACC/GCCACA	56%
	TP63	NOTCH	p.Ser38Ala	chr3:189455578	T	T/G	32%
	ARID1A	Chromatin	p.Trp1844Ter	chr1:27105920	G	G/A	19%
	KMT2D	Epigenetic	p.Thr2314Ile	chr12:49434612	G	G/A	4%

Patients	Gene	Function	Protein	Position	Reference	Tumor Variant	allele fraction
Case 10	KRAS	RTK/RAS/RAF	p.Gly12Val	chr12:25398280	GCCAC	GCCAC/GCCAA	37%
	SMARCA4	Chromatin	p.Phe1059Ser	chr19:11136983	T	T/C	27%
	RBM10	Splicing	p.Trp936Ter	chrX:47045731	G	G/A	21%
	COBL	Other	p.Glu481Ala	chr7:51098571	T	T/G	20%
	RASA1	RTK/RAS/RAF	p.Gln1034His	chr5:86686658	A	A/C	18%
	RASA1	RTK/RAS/RAF	p.Gln1035Pro	chr5:86686660	A	A/C	17%
	SMAD4	TGFb−SMAD	p.Thr197Ala	chr18:48581285	A	A/G	17%
	ATM	TP53/ Cell Cycle	p.Leu942Phe	chr11:108139322	C	C/T	17%
	MGA	Transcription	p.Lys2262Thr	chr15:42042590	A	A/C	17%
	MGA	Transcription	p.Phe665Cys	chr15:41989202	T	T/G	15%
	NOTCH2	NOTCH	p.Val1686Ala	chr1:120465015	A	A/G	15%
	TSC1	RTK/RAS/RAF	p.Leu92Phe	chr9:135801061	T	T/G	14%
	KMT2A	Epigenetic	p.Cys3962Ser	chr11:118392852	T	T/A	14%
	KMT2A	Epigenetic	p.Cys3962_Gly3963delinsTrpThr	chr11:118392854	TGG	TGG/GAC	14%
	ERBB3	RTK/RAS/RAF	p.Asp857Ala	chr12:56491678	A	A/C	13%
	TSC1	RTK/RAS/RAF	p.Pro362Leu	chr9:135786445	G	G/A	13%
	ERBB3	RTK/RAS/RAF	p.Glu448Asp	chr12:56487198	A	A/C	12%
	TSC2	RTK/RAS/RAF	p.Cys519Ser	chr16:2114384	T	T/A	12%
	ARID1A	Chromatin	p.Asn472Ser	chr1:27057707	A	A/G	12%
	FGFR2	RTK/RAS/RAF	p.Tyr780Cys	chr10:123239501	T	T/C	12%
	MET	RTK/RAS/RAF	p.Leu90fs	chr7:116339405	GC	GC/G	11%
	COBL	Other	p.Gln1004Lys	chr7:51095783	G	G/T	11%
	KMT2D	Epigenetic	p.Val4078Ala	chr12:49426255	A	A/G	10%
	KMT2D	Epigenetic	p.Val4078fs	chr12:49426258	AG	AG/A	10%
	EP300	Transcription	p.Met1989Thr	chr22:41573681	T	T/C	10%
	KMT2A	Epigenetic	p.Glu970Gly	chr11:118344783	A	A/G	7%
	COBL	Other	p.Thr403Lys	chr7:51111278	G	G/T	6%
	NOTCH2	NOTCH	p.Ala862Val	chr1:120491644	G	G/A	6%
	EPHA7	Other	p.Asp356Glu	chr6:94066691	G	G/C	5%
	KMT2D	Epigenetic	p.Thr5101Asn	chr12:49420447	G	G/T	5%
	SETD2	Epigenetic	p.Asp699Asn	chr3:47164031	C	C/T	5%
	ARID2	Chromatin	p.Met877Ile	chr12:46244537	G	G/A	5%
	MAP2K1	RTK/RAS/RAF	p.Gly77Ala	chr15:66727514	G	G/C	4%
	KMT2D	Epigenetic	p.Ala5100Pro	chr12:49420451	C	C/G	4%
	SETD2	Epigenetic	p.Arg620Ter	chr3:47164268	G	G/A	4%
Case 11	KRAS	RTK/RAS/RAF	p.Gly12Cys	chr12:25398280	GCCACC	GCCACC/GCCACA	56%
	KMT2A	Epigenetic	p.Cys3962Ser	chr11:118392852	T	T/A	4%
Case 12	ERBB2	RTK/RAS/RAF	p.Trp614Arg	chr17:37873675	T	T/C	5%
Case 13	KRAS	RTK/RAS/RAF	p.Gly12Val	chr12:25398280	GCCAC	GCCAC/GCCAA	18%
	MGA	Transcription	p.Met1236Ile	chr15:42021412	G	G/A	4%
Case 14	U2AF1	Splicing	p.Ser34Phe	chr21:44524456	G	G/A	29%
Case 15	KRAS	RTK/RAS/RAF	p.Gly12Asp	chr12:25398280	GCCAC	GCCAC/GCCAT	26%
Case 16	SMAD4	TGFb−SMAD	p.Pro298Ser	chr18:48584814	C	C/T	24%
Case 17	RBM10	Splicing	p.Arg831Leu	chrX:47044971	G	G/T	77%
	KEAP1	Oxidative	p.Glu149Lys	chr19:10610265	C	C/T	71%
	KRAS	RTK/RAS/RAF	p.Gly12Cys	chr12:25398280	GCCACC	GCCACC/GCCACA	66%
	ARID1A	Chromatin	p.Gly1469fs	chr1:27101116	A	A/AC	38%
Case 18	STK11	RTK/RAS/RAF	p.Arg297Ser	chr19:1221976	G	G/T	28%
	KRAS	RTK/RAS/RAF	p.Gly12Val	chr12:25398280	GCCAC	GCCAC/GCCAA	19%
	SMARCA4	Chromatin	p.Phe1059Ser	chr19:11136983	T	T/C	4%
Case 19	KRAS	RTK/RAS/RAF	p.Gly12Asp	chr12:25398280	GCCAC	GCCAC/GCCAT	72%
Case 20	ATM	TP53/ Cell Cycle	p.Leu2544Pro	chr11:108202607	T	T/C	59%
	KRAS	RTK/RAS/RAF	p.Gly12Asp	chr12:25398280	GCCAC	GCCAC/GCCAT	29%
	PIK3CA	RTK/RAS/RAF	p.Asn370Ser	chr3:178922340	A	A/G	21%
	KMT2D	Epigenetic	p.Asp2622Asn	chr12:49433689	C	C/T	14%
	SOX2	Transcription	p.Ser295Phe	chr3:181431032	C	C/T	5%
	SOX2	Transcription	p.Met294Ile	chr3:181431030	G	G/A	4%

Table S3. Pathways affected in IMA and NMA by mutations with allele fractions ≥ 20%.

Pathway	IMA (n=20)	NMA (n=43)	*p* value
RTK/RAS/RAF	16 (80.0%)	30 (69.8%)	0.394
TP53/ Cell Cycle	5 (25.0%)	15 (34.9%)	0.433
Transcription	0 (0.0%)	3 (7.0%)	0.226
NOTCH	0 (0.0%)	3 (7.0%)	0.226
Epigenetic	1 (5.0%)	1 (2.3%)	0.573
Oxidative	3 (15.0%)	4 (9.3%)	0.503
Chromatin	3 (15.0%)	1 (2.3%)	0.055
Splicing	4 (20.0%)	7 (16.3%)	0.717
Wnt	1 (5.0%)	0 (0.0%)	0.139
TGFb-SMAD	3 (15.0%)	2 (4.7%)	0.157
Axon guidance	0 (0.0%)	1 (2.3%)	0.492
Other	0 (0.0%)	1 (2.3%)	0.492

Table S4. Sequencing targets.

No	Gene symbol	Chromosome	Number of Amplicons	Total Bases	Covered Bases	Overall Coverage
1	AKT1	chr14	26	1573	1497	95%
2	AKT2	chr19	27	1576	1543	98%
3	AKT3	chr1	30	1624	1624	100%
4	ARID1A	chr1	76	7058	6023	85%
5	ARID1B	chr6	75	6950	5965	86%
6	ARID2	chr12	71	5718	5643	99%
7	ASCL4	chr12	5	532	382	72%
8	ATM	chr11	147	9791	9439	96%
9	BRAF	chr7	37	2481	2224	90%
10	CDKN2A	chr9	9	962	612	64%
11	COBL	chr7	48	4151	3977	96%
12	CREBBP	chr16	96	7639	7071	93%
13	CTNNB1	chr3	32	2486	2486	100%
14	CUL3	chr2	42	2561	2495	97%
15	EGFR	chr7	60	4189	4135	99%
16	EP300	chr22	90	7555	7182	95%
17	EPHA7	chr6	44	3175	3154	99%
18	ERBB2	chr17	57	4080	3808	93%
19	ERBB3	chr12	59	4440	4374	99%
20	FGFR1	chr8	41	2825	2816	100%
21	FGFR2	chr10	43	2910	2842	98%
22	FGFR3	chr4	34	2752	2215	81%
23	FOXP2	chr7	36	2487	2469	99%
24	HRAS	chr11	11	683	683	100%
25	KEAP1	chr19	24	1925	1845	96%
26	KMT2D	chr12	192	17154	15854	92%
27	KRAS	chr12	10	737	681	92%
28	MAP2K1	chr15	18	1292	1239	96%
29	MET	chr7	59	4427	4396	99%
30	MGA	chr15	110	9428	9345	99%
31	MLL	chr11	144	12279	11875	97%
32	NF1	chr17	136	9161	9023	99%
33	NFE2L2	chr2	23	1868	1826	98%
34	NOTCH1	chr9	99	8008	7078	88%
35	NOTCH2	chr1	101	7809	7539	97%
36	NRAS	chr1	9	610	610	100%
37	PIK3CA	chr3	50	3407	3282	96%
38	PTEN	chr10	18	1302	1223	94%
39	RASA1	chr5	55	3412	3216	94%
40	RB1	chr13	55	3057	2902	95%
41	RBM10	chrX	48	3228	3079	95%
42	RIT1	chr1	13	771	771	100%
43	SETD2	chr3	91	7905	7663	97%
44	SLIT2	chr4	76	4972	4854	98%
45	SMAD4	chr18	24	1769	1715	97%
46	SMARCA4	chr19	74	5399	5055	94%
47	SOX2	chr3	9	964	883	92%
48	STK11	chr19	23	1392	1343	97%
49	TP53	chr17	22	1383	1351	98%
50	TP63	chr3	34	2360	2227	94%

51	TSC1	chr9	49	3705	3603	97%
52	TSC2	chr16	92	5834	5677	97%
53	U2AF1	chr21	15	880	870	99%

© 2018 by the authors. Licensee MDPI, Basel, Switzerland. This article is an open access article distributed under the terms and conditions of the Creative Commons Attribution (CC BY) license (http://creativecommons.org/licenses/by/4.0/).
